# Assessment Model for Rapid Suppression of SARS-CoV-2 Transmission under Government Control

**DOI:** 10.3390/tropicalmed7120399

**Published:** 2022-11-25

**Authors:** Lihu Pan, Ya Su, Huimin Yan, Rui Zhang

**Affiliations:** 1 School of Computer Science and Technology, Taiyuan University of Science and Technology, Taiyuan 030024, China; 2 Institute of Geographic Sciences and Natural Resources Research, Chinese Academy of Sciences, Beijing 100101, China

**Keywords:** multi-agent modeling, public health security, SARS-CoV-2 transmission, urban outbreak prevention and control, virus prevention and control strategies

## Abstract

The rapid suppression of SARS-CoV-2 transmission remains a priority for maintaining public health security throughout the world, and the agile adjustment of government prevention and control strategies according to the spread of the epidemic is crucial for controlling the spread of the epidemic. Thus, in this study, a multi-agent modeling approach was developed for constructing an assessment model for the rapid suppression of SARS-CoV-2 transmission under government control. Different from previous mathematical models, this model combines computer technology and geographic information system to abstract human beings in different states into micro-agents with self-control and independent decision-making ability; defines the rules of agent behavior and interaction; and describes the mobility, heterogeneity, contact behavior patterns, and dynamic interactive feedback mechanism of space environment. The real geospatial and social environment in Taiyuan was considered as a case study. In the implemented model, the government agent could adjust the response level and prevention and control policies for major public health emergencies in real time according to the development of the epidemic, and different intervention strategies were provided to improve disease control methods in the simulation experiment. The simulation results demonstrate that the proposed model is widely applicable, and it can not only judge the effectiveness of intervention measures in time but also analyze the virus transmission status in complex urban systems and its change trend under different intervention measures, thereby providing scientific guidance to support urban public health safety.

## 1. Introduction

Since the outbreak of the worldwide COVID-19 pandemic in 2020, the growth of local outbreaks has been controlled to some extent by the aggressive preventive and control measures implemented by the Chinese government and the concerted efforts of the whole nation [1]. However, due to the emergence of various mutant strains of SARS-CoV-2 that are more infectious, with a significantly shorter transmission interval and higher viral load than the original coronavirus, the number of confirmed cases and deaths caused by coronavirus disease has again continued to rise, and there has even been a trend toward “breakthrough infection” among COVID-19 vaccine recipients in some countries. SARS-CoV-2 variants have been transmitted worldwide, and the need to prevent and control the spread of the epidemic has again been highlighted. Therefore, in the process of global cooperation to combat the epidemic, it is important to construct a more targeted SARS-CoV-2 transmission model in response to the changing virus transmission characteristics and the constantly improving epidemic prevention and control measures.

Traditional epidemic transmission methods are mainly mathematical, generally using differential equations to describe the relationship between changes in individual states, analyzing the characteristics of equilibrium solutions and the relationship between parameters, and obtaining the transmission pattern of infectious diseases in the population [2]. Currently available simulation models of novel coronavirus pneumonia are mainly based on infectious disease dynamics models. Common infectious disease dynamics models are classified according to the type of infectious disease as SI, SIR, SIRS, SEIR models, etc. [3]. They are used to divide the population into different groups according to their different health states, and differential equations are used to describe the dynamic evolution process of the number of individuals among different groups. For example, the SEIR model contains four groups of susceptible, exposed, infected, and recovered individuals, and the relationship between them is represented by the conversion rate in the differential equation [4]. The traditional mathematical models of infectious diseases make some assumptions and simplifications on the complex process and factors of epidemic transmission, which can theoretically analyze and describe the macroscopic pattern of the spread and diffusion of novel coronavirus pneumonia, but at the same time, it also brings limitations and make it difficult to describe the complex process of epidemic transmission in detail [5]. First of all, the assumption of uniform population mixing makes it difficult for the mathematical model of epidemic transmission to describe the differences of individual micro-behavior and interaction. Secondly, fewer parameters make it difficult to describe the wide variety of factors associated with the spread of an epidemic, especially the complex behavior of humans. Finally, the parameters assigned to the mean in the differential equation have difficulty describing the heterogeneity in the epidemic transmission process, such as the heterogeneous transmission capacity of infectious individuals and the heterogeneous time scale of disease course development [6].

Based on the above limitations, researchers in China and other countries have improved various intervention assessment models for repeated outbreaks and to expand the mathematical model to predict the development of epidemic. J Huang et al. [7] developed a global epidemic prediction system by improving the SIR model combined with temperature and humidity. In the system, assuming that there is no immune difference between individuals and the total population during the outbreak period is constant, the total population of each country is divided into the susceptible population (S), the infected population (I), and the cured and dead population (R). Leontitsis A et al. [8] developed the SEAHIR model by improving the SEIR model and proposed the use of a six-segmented model for current or future epidemics. Teslya A et al. [9] developed a COVID-19 regional transmission model to predict the incidence, maximum number of diagnoses, and time to maximum number of diagnoses. Ayele W et al. [10] evaluated and predicted epidemics at different time points in a given region using the SEIR model. Yang Z et al. [11] introduced the input/output flow on the basis of the SEIR model and divided the original propagation speed parameters into two flow directions based on the traffic data between provinces and cities, so that the population changes during the model deduction process are more realistic. Most of these models were developed by improving the existing mathematical models of epidemic transmission, which still have limitations. (1) Mathematical methods have insufficient research on the dynamic evolution of the infectious disease transmission process, without considering the mobility of the population; (2) the spread behavior is closely related to the individual behavior of the crowd, and mathematical models generally assume that the individual behavior attributes are the same, and It is not evenly mixed in the crowd and lacks the ability to describe individuals at the micro level, and it is difficult to reflect the influence of human heterogeneous social activities and flow patterns on the communication process [12]; (3) with the increase of constraints, the complexity of the model increases, and the form of the solution is complex and cannot be analyzed [13]; (4) it is useful to predict the spread of the original novel coronavirus over time without considering stochasticity, spatial effects, contact mode, interaction behavior, and adaptive changes as the environment changes [14]. In fact, the previous control measures and prevention strategies for the original novel coronavirus are no longer fully suitable for the transmission characteristics of the current or even future mutated strains. The effectiveness of existing inactivated vaccines at blocking infection by mutated strains has also been compromised.

The interactions between human self-control and autonomous decision-making behavior and the spatial environment influence the transmission of the epidemic. With the continuous contact between individuals in the artificial society and the simulation of SARS-CoV-2 transmission based on individual contact, a macroscopic epidemic transmission will be presented and even a new emergence phenomenon may occur. A multi-agent system is an autonomous agent organization that interacts with each other in a shared environment [15]. Agents are heterogeneous entities used to model different individuals [16]. The environment is a virtual representation of the region where the agent is located. The interaction between agents and between agents and the environment is simulated through interactive behavior [17]. Among them, the mobile interaction behavior of agents is often more suitable for simulating SARS-CoV-2 propagation dynamics. Therefore, in the present study, multi-agent modeling theory was combined with the current epidemic situation and virus transmission characteristics to construct a rapid SARS-CoV-2 transmission suppression assessment model under government control, mainly considering the role of the natural environment, individual attributes, and the interactions of people in the process of epidemic transmission. It is not only suitable for the original coronavirus but is also suitable for the variant strain of the coronavirus. It takes the human individual as the modeling object and abstracts the human being as an autonomous agent [18]; the heterogeneous random behavior of individuals can be described in detail, and agents can be classified according to the heterogeneity of the population. Computer technology is used to build individual models of crowds using agents, define the structure of social relationships and contact behavior patterns between individuals, and combine mathematical models to describe intelligences’ behavior and interaction rules. At the same time, GIS technology is used to describe geospatial information to simulate the interaction between the agent and the spatial environment. To a certain extent, it reproduces the situational, behavioral, and evolutionary characteristics of epidemic spread and prevention and control and then carries out simulation to realize the emergence of group complex behavior.

In this model, the environmental model is refined, the study area is divided, and the population distribution and flow speed are affected by different building groups, climatic conditions, and road traffic. The reactive agent is used to establish an individual population model, and the attributes, behaviors, and interactions of agents are described randomly and heterogeneously. According to the external information input, agents make judgments and update their own decision-making and output behavior and then affect the environment. Intervention measures such as nucleic acid testing, vaccination, and tracking close contacts are introduced, and hospital agents are introduced to affect the time of treatment and the development of the disease course through distance and medical resources. At the same time, the government can sense the spread of the epidemic in real time and adjust the response level and prevention and control policies for major public health emergencies. Based on the dynamic feedback mechanisms between humans and the spatial environment, different intervention measures can be simulated for implementation at different times under government control, and different intervention strategies are provided to improve disease control methods and to analyze the effectiveness of SARS-CoV-2 prevention and control.

## 2. Multi-Agent Model Construction

### 2.1. Overview of the Model

Based on multi-agent simulation techniques, an evaluation model for rapidly suppressing SARS-CoV-2 transmission under government control is proposed for simulating changes in the states of resident agents and the transmission and prevention and control of the virus under different scenarios. The model was developed by the research team using the Repast Simphony 2.8.1 (Argonne National Laboratory, Lemont, IL, USA) simulation modeling tool and written in the Eclipse development platform based on the JAVA language. ArcGIS (Esri, Redlands, CA, USA) is used to rasterize real geographic data to provide operational data for the model. The model can predict the developmental trend of SARS-CoV-2 transmission and explores the inhibitory effects of different SARS-CoV-2 prevention and control measures. The model can predict and analyze different possible scenarios to provide decision support for urban virus prevention and control. The main idea of the model involves abstracting the real urban system and the SARS-CoV-2 transmission process to simulate the changes in virus transmission and health status during the movements and contacts of individual agents. The model simulates the decision-making behaviors of government, university, station, and shopping-area agents in order to adjust the corresponding level of prevention and control. The model can be used to analyze the impacts of population movements and contacts on SARS-CoV-2 transmission through spatial collaboration among agents to further analyze the SARS-CoV-2 transmission and infection process and to predict its general development trend under different prevention and control measures. The general steps for building an evaluation model for rapidly suppressing SARS-CoV-2 transmission under government control are as follows: (1) Extraction and generalization of the main model elements in the real artificial social environment system to identify the main heterogeneous entities in the SARS-CoV-2 transmission and prevention and control system. (2) Identification of the attributes and behaviors of each type of agent based on the characteristics and roles of the entity objects in the artificial society. (3) Determining the interactions among the various types of agents based on the available real data to assess the effects of multiple factors over time and in space to model the rapid suppression of virus spread under government control. (4) Conducting simulation experiments using the constructed simulation model.

**Figure 1:**
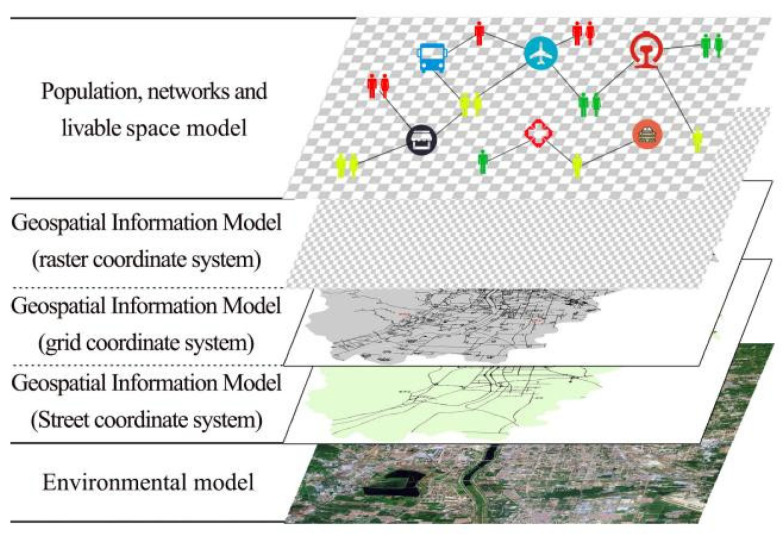
Model hierarchy.

The rapid suppression SARS-CoV-2 transmission assessment model maps the real urban environment and movements of people to a computer system, which can be divided into five levels, as shown in Figure 1. The first level represents the environmental composition of the real artificial society, including the ecological environment, roads and traffic, environmental climate, and distribution of building clusters. The second level establishes the street coordinate system based on geospatial information. The geospatial environment for this model is based on the six municipal districts under the jurisdiction of Taiyuan City: Yingze District, Xinghualing District, Wanbailin District, Jiancaoping District, Jinyuan District, and Xiaodian District. In particular, the streets are selected to divide the urban geospatial space according to the highways and primary urban roads in Taiyuan city. The third level establishes a grid coordinate system based on geospatial information to determine the spatial location of each entity. The grid improves the level and efficiency of urban management. The Urban Planning Bureau relies on information technology and follows relevant national standards, industry standards, and local standards to implement grid division for the whole city in order to promote the in-depth development of urban grid management and information construction [19]. The fourth level is based on geospatial information to establish a raster coordinate system corresponding to the real artificial social environment mapping, wherein the raster size of each unit is 244 m × 244 m. The fifth level represents a spatial model of the population organization network and virus propagation, including the individual agents in the population, various elements in the city in the artificial society, states and behavior of various agents, and the correlations and interactions among various agents.

### 2.2. Construction of the Spatial Environment Model

The spatial environment in the model is the space where the activities of various types of agents occur in the model, and it is also the spatial carrier of various elements of the SARS-CoV-2 transmission and prevention and control system. In addition to population flow and interpersonal contact, environmental factors can affect the survival and transmission of the virus [20]. The spatial environment in the rapid suppression of SARS-CoV-2 transmission under government control assessment model maps the real spatial environment of the real artificial society, which requires the discrete treatment of the geographic space of the city according to the following two levels. The first level is the natural geographic environment, which mainly includes the geographic space and urban climate factors. The second level is the social environment, which mainly includes the population distribution, public facilities distribution, and policy information.

#### 2.2.1. Geospatial Discretization

**Figure 2:**
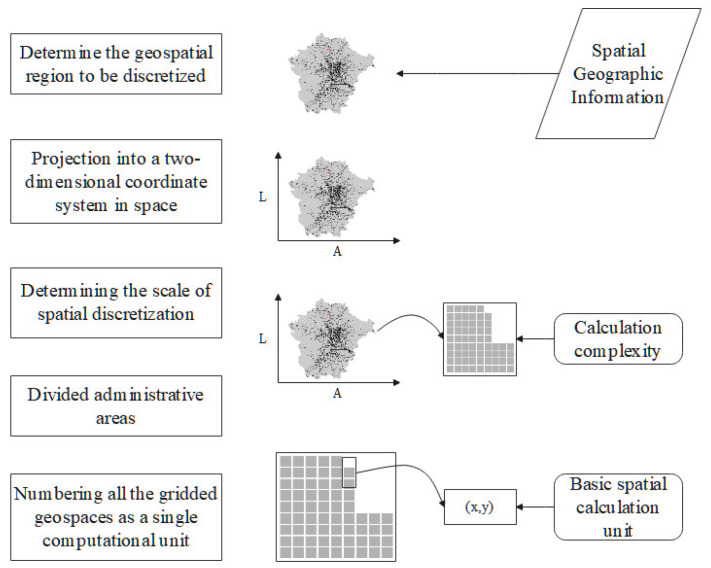
Geospatial discretization process.

In the simulation of SARS-CoV-2 transmission, prevention, and control, the main behaviors of the resident agents involve movements and contacts through autonomous decision-making, and their willingness to move is a composite indicator. In the mobility process, it is necessary to search for the environmental entities, and their willingness to move is influenced by the calculated distances. The traditional method for locating spatial points based on latitude and longitude coordinates can clearly characterize the geographical locations of environmental entities on a map, but it is not conducive to searching for environmental entities under the satisfaction of constraints. Therefore, the geographic space in the city is discretized to optimize the computational efficiency of the spatial search algorithm, and the basic idea of this algorithm is shown in Figure 2.

The specific steps are as follows: (1) Determine the geospatial region for discretization and extract its boundary outline from ArcGIS. (2) Project the region onto a two-dimensional latitude and longitude coordinate system. (3) Determine the scale of spatial discretization according to the computational complexity that the computational resources can handle. (4) Discretize the model area within the latitude–longitude coordinate system according to the determined discretization scale to obtain the gridded geospatial map. (5) Number all of the gridded geospaces as single computational units for later computational processing, with each computational unit represented by a set of horizontal coordinates (x,y) where x and y are integers. According to the distance metric for the discretized geospatial space, adjust the coordinates of the square projection where the central coordinate point is located $\left(x_{c},y_{c}\right).$ The set of squares at a distance of d km is shown in Formula (1).

(1) $$\begin{array}{c} (x_{c}+d,y),y\in \left[y_{c}-d,y_{c}+d\right]\\ (x_{c}-d,y),y\in \left[y_{c}-d,y_{c}+d\right]\\ (x,y_{c}+d),y\in \left[x_{c}-d,x_{c}+d\right]\\ (x,y_{c}-d),y\in \left[x_{c}-d,x_{c}+d\right] \end{array}$$

#### 2.2.2. Physical Geography

1. Geographical space

The geographical space refers to the geographical area studied using the model. The SARS-CoV-2 transmission and prevention and control social simulation model treated Taiyuan, an important city in central China, as the research object, and the environmental model had relatively high granularity. The simulation study was conducted for different population densities, locations of public facilities, and natural geographical environments in the six municipal districts of Taiyuan. The 1:13 land in the urban area of Taiyuan City was selected, where the geospatial coordinate system was established with the raster technique using 1032 × 959 raster blocks as the geographical space for the model.

2. Climate factors

According to global epidemiological analysis, changes in temperature and relative humidity due to changes in climate can have significant impacts on the survival and spread of viruses. In the present study, the average temperature per month in Taiyuan was compiled for 10 years. The climate factor defined in the model is a random number that fluctuates within a certain range, and this range was determined by comparing the existing temperature data with the time that the model was run, thereby ensuring the randomness and reasonableness of the impact of climate in different months, which is more in line with the realistic climate environment for the survival of urban residents. The numbers of confirmed cases of pneumonia due to SARS-CoV-2 were observed under different climatic temperatures to study the developmental trend of the epidemic by conducting simulations of the effects of different temperatures on the prevention and control of the epidemic.

#### 2.2.3. Social Environment

The model mainly considers two types of environments: the public facilities distribution environment and population distribution environment. In this model, the distributions of public facilities affect the mobility and aggregation of the population, wherein the railway station agent, airport agent, and agent around the business district will increase the willingness of the population flow and issue different prevention and control measures according to the current epidemic prevention and control level. These changes affect the transmission speed of the SARS-CoV-2 virus according to the current population density and prevention and control measures via interactive feedback between multiple agents. The healthier agents in the model in the initial state are distributed according to the proportions of the real population distributions in the six municipal districts under the jurisdiction of Taiyuan city in 2020. The public facilities in the social environment include the government, hospitals, airports, stations, shopping areas, and universities, and their geographical locations are mapped onto the gridded geographic space according to the actual latitude and longitude coordinate system.

### 2.3. Definition of an Agent

#### 2.3.1. Agent Types and Properties

The agent-based rapid suppression of SARS-CoV-2 transmission assessment model employs a bottom-up modeling approach to observe the complex phenomena that emerge from various complex systems in real life. The model can provide detailed descriptions of the micro-attributes and behaviors of individual populations, as well as the micro-processes related to epidemic transmission, and allow the emergence of complex macro-social phenomena through the behaviors and interactions among individuals. In order to better describe the heterogeneity and randomness of the epidemic transmission process, five categories of resident agents are abstracted, with different states comprising healthy, latent, close contact, quarantined, and confirmed individuals, as well as government, hospital, airport, railway station, bus station, university, and business district agents, and each have their own attributes and behaviors.

The attributes of the resident agents include their current location, age, current ambient temperature, personal willingness to move, target location, arrival status, whether vaccinated, whether wearing a mask, moment of close contact with the pathogen, time of becoming an latent agent, time of isolation, time of nucleic acid testing, number of nucleic acid tests, time of onset, time of diagnosis, time of hospitalization, and severity of illness. In particular, the healthy-person agent is the most basic individual unit in the model because the residents are healthy in their initial state. The quarantined agent and confirmed agent do not have the ability to move because they are controlled by isolation. The main attributes of the hospital agent include the current location, remaining effective number of beds, hospital level, amount of medical resources, and response time for admitting the confirmed agent. The attributes of the university agent include the current location, current prevention and control level, whether the school is closed, and whether it is confirmed that people entering the campus are wearing masks, whether the trip code and health code are green codes, whether the nucleic acid test proves negative, and the vaccination records. The main attributes of the train station agent, bus station agent, and airport agent include the location, current level of prevention and control, and whether it is confirmed that people entering the city are wearing masks, whether the trip code and health code are green, whether the nucleic acid test certificate is negative, and the vaccination record. The main attributes of the government agent include the name of the government, level of response to major public health emergencies, and the adjustment of prevention and control policies.

#### 2.3.2. Design of Agent Interactions and Information Transfer

**Figure 3:**
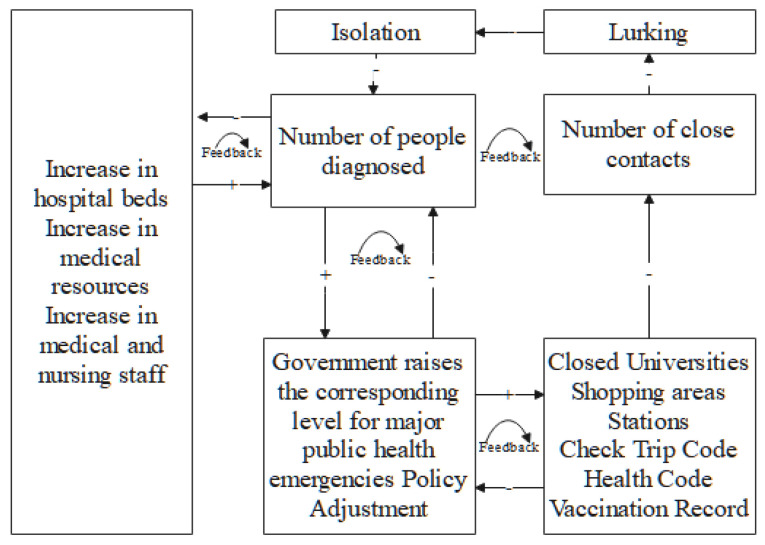
Interactions and feedback among various agents.

A multi-agent system comprises a series of interacting agents. Interactions to transfer information between agents and agents and between agents and the spatial environment are core parts of the assessment model for the rapid suppression of SARS-CoV-2 transmission. Communication between different agents follows certain rules to form movement trajectories and make behavioral decisions and state transitions by sensing changes in the surrounding environment, which lead to self-organization and self-learning mechanisms. The complexity of the artificial society determines that many types and numbers of agents constitute the system, and the functions and structures of various types of agents are complex, with nonlinear relationships and complex interactive behaviors. As shown in Figure 3, “+” indicates a positive correlation between two types of agents, and “−” indicates a negative correlation between two types of agents. Four feedback loops are identified between different agents. The first is the interactive feedback from the number of confirmed agents and hospital agents. The second is the interactive feedback from the number of confirmed agents and government agents. The third is the interactive feedback from the government agents and universities, business districts, and stations. The first three feedback loops combine with the resident agents in different states to form another complete closed loop. When building the model, it was found that the agents and spatial environment in each time sequence affected changes in the epidemic. All types of agents update their states and make responses in real time during each simulation time step to finally complete the interactive feedback between agents and between the agents and the spatial environment.

#### 2.3.3. Analysis of Agent Behavioral Activity

The complex behavioral activities of different agents in the model are determined according to complex phenomena that emerge from various complex systems in real life. The different agents in the model follow certain rules and carry out periodic activities based on changes on time and space and the interactions between different agents. These behavioral activities are a true reflection of the realistic SARS-CoV-2 transmission and prevention and control process. The specific behaviors and descriptions of different agents are shown in Table 1.

#### 2.3.4. Agent Behavior Rule Design

1. Design of behavioral rules for resident agents

**Figure 4:**
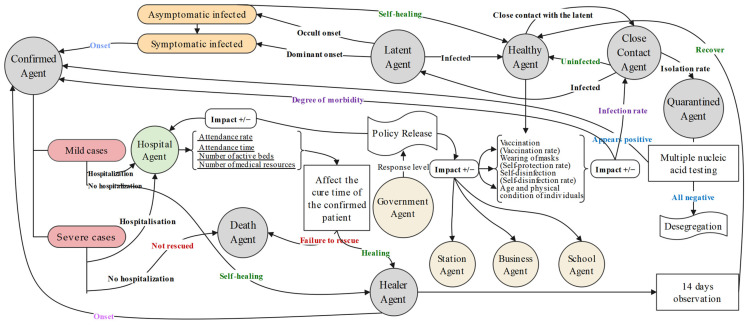
State transition diagram for resident agents.

The behavioral decisions of the various types of agents in the model are the main processes that affect the SARS-CoV-2 virus transmission process [21,22]. In particular, resident agents are variables in the simulation process, wherein they make autonomous decisions to generate certain behaviors by perceiving the surrounding entities and the prevention and control policies of the current environment. Their states will change with time and space, and they will take certain personal prevention and control measures autonomously [23]. The behavioral rules of the resident agents mainly include the mobile behavior, contact propagation, and state transition rules [24]. As shown in Figure 4, the state transition rules of the resident agent are improved in this model. Initially importing resident agents are judged to be healthy, and importing latent agents at stations and airports and healthy agents have different probabilities of infection risk depending on their mobility needs and the adoption of different prevention and control measures. The movements of the latent agents during the incubation period cause healthy agents in close contact with them to become close contacts and then to become confirmed agents after the onset of the disease. Close-contact agents will become quarantined agents, mobile latent agents, or uninfected mobile close-contact agents at a rate based on the current level of prevention and control issued by the government agents, and the prevention and control measures taken prior to becoming a close-contact agent. The quarantined agent will have a certain probability of becoming a confirmed agent based on the individual control measures taken previously and will undergo regular nucleic acid testing during the quarantine period, after which they will become a healthy agent if all tests are negative or a confirmed agent if all tests are positive; they may also die during the quarantine period without receiving timely treatment. Confirmed persons will have a certain probability of becoming mildly ill, seriously ill, dead, or cured due to the duration of treatment depending on the age of the resident agent, any pre-exposure precautions, the adequacy of medical resources, and the timeliness of treatment.

2. Government Agent Emergency Response Behavioral Rules

The government agent can adjust the response levels and prevention and control policies for major public health emergencies based on the current number of confirmed agents and quarantined agents according to the specific classification criteria shown in Table 2. According to the different emergency response levels, a government agent will also increase its efforts to publicize SARS-CoV-2, to implement policies such as free vaccination and uniform vaccination by units or industries, and to improve the conditions of vaccination facilities to influence the vaccination rate of the population’s agent and further build up a population immune barrier in the resident agents to interrupt the spread of the novel coronavirus among the population.

In particular, the level IV emergency response involves the small-scale wearing of masks and disinfection, patient treatment, and medical observation. The level III emergency response involves the isolation of close contacts and research into vaccines and other solutions. The level II emergency response involves large-scale tracking and isolation, the small-scale suspension of work and schools, and the organization of small-scale vaccination. The level I emergency response includes large-scale vaccination and the suspension of work and schools.

3. Rules of conduct for interventions by agents in stations, shopping areas, and universities

Stations, shopping areas, and university agents will adapt their individual interventions to the conditions in the municipal district where they are located and to the government agents’ current response levels and prevention and control policies for major public health emergencies. Resident agents can perceive the stimulation of surrounding entities and the environment and instinctively make their own responses. The willingness to flow is expressed by $Flow_{i}$. The willingness to flow of resident agents increases in the areas around stations and airports and among business-district agents, and the willingness to flow of resident agents decreases in the areas around university agents. Adjusting the government agents’ response levels to major public health emergencies and prevention and control policies will affect the willingness to flow of resident agents with a certain weight. As shown in Formula (2), the value of the personal mobility intention is the sum of the average mobility intention and random mobility intention.

(2) $$Flow_{i}=\sigma \times Rmove_{i}+\mu$$

In the formula, $Rmove_{i}$ is a random number from a Gaussian distribution generated according to the weight of flow intention. The individual flow intention $Flow_{i}$ follows a Gaussian distribution [25]. The fluctuation in the personal mobility willingness value is described by σ, and μ determines the position of the Gaussian distribution. In the iterative modeling process, the individual flow intention $Flow_{i}$ determines whether the resident agent moves, and it moves when this value is positive. As shown in Formula (3), the contact transmission rate of each latent agent will change with the speed of the crowd’s flow.

(3) $$f(contacts_{num})=\frac{1}{1+e^{-contacts_{num}/2}}$$

where $contacts_{num}$ represents the number of people who have been contacted by the latent agent at present, and $f(contacts_{num})$ represents the contact transmission rate. The number of infections caused by the latent agent during the flow process $Infected_{Num}$ is shown in Formula (4).

(4) $$N_{i}=(f(contacts_{num})\times Flow_{i})\times contacts_{num}$$

The station and airport agents will make the corresponding policy adjustments according to the current number of resident agents in the surrounding area, as well as the flow speed and frequency, and track the information for passengers. When the flow record for the confirmed agent appears, the time and track of the confirmed agent flowing in the station will be tracked, and the states of resident agents who have been in contact within a specified time range will be converted into close-contact agents. The corresponding inbound and outbound interventions will be adjusted according to the current response level of the government. When the response level is above level IV, the inbound passengers need to provide travel code, health code, and vaccination records, and the flow rate of people within the specified range slows down. A negative nucleic acid test certificate should be provided for level II and above, and the station mobility rate and number of people will be reduced accordingly.

Universities and business-district agents can perceive the response level of the current government and the policies issued, adjust their intervention measures, and control the flow of people. The adjustment rules for the intervention measures of university agents are as follows. At government response level I, both universities and business-district agents take closing measures, and the crowd within their designated range will not flow. At government response level II, university agents still take closing measures, business-district agents take measures to slow down the flow rate of people within the specified range, and mobile resident agents take measures to check travel codes, health codes, vaccination records, etc. At government response level III, the university agents still take level II measures; the business district agent take measures to restore the crowd flow rate to normal within the specified range; and the moving crowd takes measures to check the itinerary code, health code, vaccination record, etc. At government response level IV, both universities and business-district agents check whether the mobile resident agents within the specified range are wearing masks, and the flow rate of people wearing masks returns to normal.

## 3. Multi-Agent Based Simulation for the Rapid Suppression of SARS-CoV-2 Propagation

### 3.1. Operational Flow of the Simulation System

**Figure 5:**
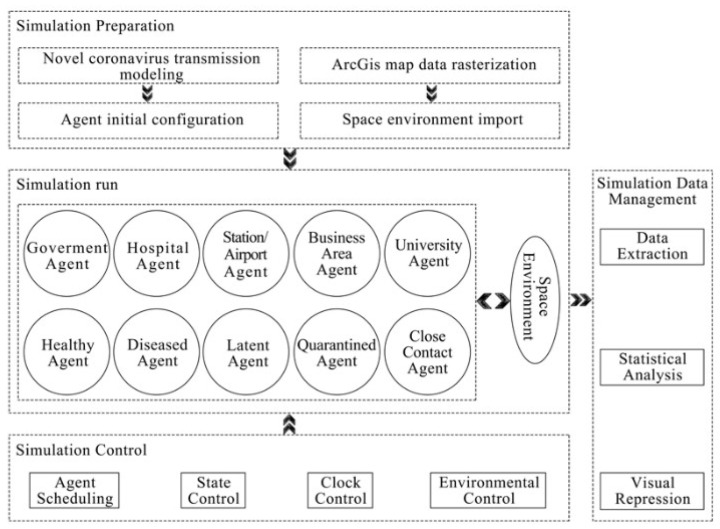
System operation process.

The behaviors of human society in major public health emergencies follow simple local microscopic rules and complex overall macroscopic mechanisms with nonlinear characteristics. The use of computer modeling and simulation techniques to build a model with strong similarity to urban social systems is important for analyzing the spread, prevention, and control of SARS-CoV-2 in cities. The multi-agent-based government-controlled rapid SARS-CoV-2 propagation suppression assessment model developed in this study can be divided into three phases: the simulation preparation phase, the simulation operation phase, and the simulation data management phase. The operational flow of the model is shown in Figure 5.

The simulation preparation stage involves the initial configuration of the spatial environment in the model, as well as information regarding the interactions between the agents by providing actionable data. The initialization of the spatial environment data involves converting the ArcGIS map data for Taiyuan city into ASCII format, which can be recognized by the model and used as the operating environment for various types of agents in the model. The agent data are initialized as a series of characteristics of agents according to the corresponding data, which are visualized, and this mainly involves marking government, hospital, university, station, and business-district agents in the model according to real geographic data and dividing the raster near the area to which they belong. Different intervention measures are then taken to prevent virus transmission when resident agents flow to this area. Based on the information described above, the healthy-person agents are distributed at different densities according to the proportions of the population in different municipal districts and important geographical locations, and the latent person agents are introduced into the areas with large flows near the station agents.

The simulation operation stage is crucial for model construction. In each simulation time step, as the environmental data are updated, various agents follow different behavior rules for interactive feedback and their own state transformation to form a self-organization and self-learning mechanism. The corresponding decisions and behaviors are produced, such as the number quarantined, close contacts, death statistics, number of cured people, and the corresponding intervention measures and the response level of the government are changed according to the data. The interaction feedback between agents in the model and the interaction feedback between the agents and space environment constitute a complex evolving spatiotemporal system.

The simulation data management phase ensures the stable operation of the system. The module control unit ensures the orderly operation of the various components of the model. Agent scheduling allocates resources to the agents running in each simulation time step. State control is mainly for controlling the state changes of the various types of agents and the environment in the model. Clock control is mainly responsible for changing the number of simulation steps, and environment control is mainly responsible for the import, change, and export of the environment in the model. These four control mechanisms work together to extract, store, and statistically visualize the data as the model runs. The data analysis module extracts the data from the model runs and then presents them statistically in graphical form.

### 3.2. Simulation Model Implementation

In the present study, six districts of Taiyuan City, Shanxi Province, were selected as the research area, and simulations were performed according to the real data. The main parameters in the model are as follows. The total population of the Taiyuan district was set to 3.02159 million [26]. According to data from the seventh Taiyuan population census in 2020, the age composition of the population was set as 15.55% for 0–14 years old, 68.34% for 15–59 years old, and 16.11% for 60 years old and above. According to data regarding the national complete vaccination rate on 17 March 2022 published by the National Health Commission of the People’s Republic of China, the vaccination rate and mortality was set at 87.85% and the case fatality rate at 2.1%. The transmission rate was set at 76%, and the latency was set at 4.4 days [27]. The mean time of death was set at 16.7 days [28]. The average cure time was set at approximately two weeks for mild diseases and 3–6 weeks for severe and critical diseases [29]. The wearing of masks could reduce the infection rate by 80% [30]. Vaccination reduced the infection rate to 95% [31]. According to the Novel Coronavirus Pneumonia Prevention and Control Program (Eighth Edition), the average number of nucleic acid detection tests in an isolation period was set at four. Novel coronavirus pneumonia is pneumonia caused by SARS-CoV-2. The number of medical staff and beds were set for 11 hospitals in Taiyuan city area with level 3a and above, which had the ability to treat the new type of pneumonia due to SARS-CoV-2. Each hospital provided 15% of the total number of beds and the total number of medical staff in accordance with the corresponding medical carrying capacity for the novel pneumonia due to SARS-CoV-2. According to the new management standard for setting up hospitals for treating the novel pneumonia due to SARS-CoV-2, the mild patients had to achieve a health care ratio of 1:2.5, with a bed protection ratio of 1:1, and the severe patients had to achieve a medical care ratio of 1:3, with a bed protection ratio of 1:6.

**Figure 6:**
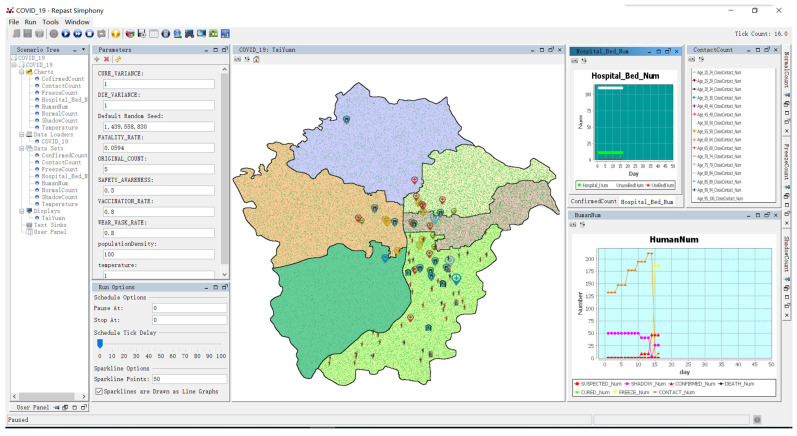
Model operating interface.

The operating interface of the model is shown in Figure 6. During the operation of the model, real-time status data statistics were used for resident agents of different ages. According to the different initial population and infrastructure distributions in different municipal districts, the numbers of healthy people with different ages, quarantined people, close contacts, confirmed cases, deaths, and cured people were the outputs. The map part of the interface was the corresponding model space environment with the current operating state for virus transmission and prevention and control. Different landmarks represented the locations of different spatial agents, and the humanoid icons with different colors moving on the map represented resident agents in different states.

The model simulated the virus transmission process and evaluated the prevention and control effects of different intervention strategies under the control of the government based on the complex process of interaction between the change in the state of the resident agents and behavioral decisions, the level of government response to major public health emergencies, the adjustment of intervention policies of different organizations, and the allocation of medical resources, in order to provide a reference for government decision-making regarding urban virus prevention and control.

### 3.3. Model Validation

**Figure 7:**
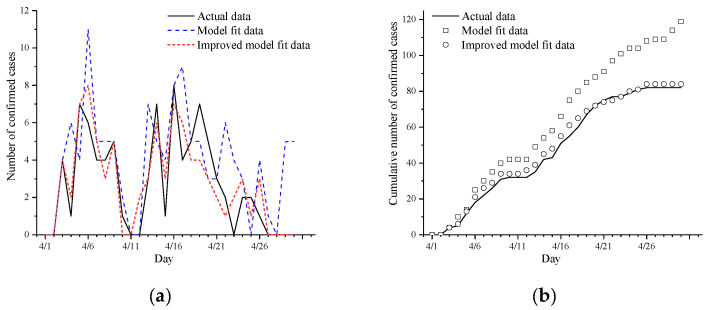
Model fitting results: (**a**) daily new confirmed fitting results for Taiyuan; (**b**) daily cumulative confirmed fitting results for Taiyuan.

In order to verify the accuracy of the model and parameters, a comparison was made with the existing COVID-19 virus prevention and control simulation model [24] in Taiyuan. The real data for the local confirmed cases in Taiyuan in April 2022 were compared with the simulated data, and the models were all adjusted to uniform parameters according to the real data and imported into the model for inversion. The results of the comparison are shown in Figure 7.

As shown in Figure 7b, with the advance of the simulation time step, the deviation between the cumulative confirmed number predicted by the existing COVID-19 virus prevention and control simulation model in Taiyuan and the real value gradually increases. The reason is that most of the transmission of COVID-19 is in crowded areas, and the flow willingness and speed of the crowd will also be affected to a certain extent according to the current level of prevention and control. Furthermore, the large-scale vaccination with COVID-19 vaccine, nucleic acid testing, and tracking close contacts have affected the traditional law of epidemic spread. However, the existing models do not take into account the willingness and velocity of resident agent flow under different levels of government control, and the target points for the flow of resident agents are randomly determined and not targeted. Existing models also do not divide the model space environment. The entire geographical space of Taiyuan is used as the simulation area, and the sparsely populated mountainous areas are not considered. The movement of people is very small, and the risk of virus transmission is small. At the same time, the state of the latent agent in the existing model is directly transformed into a confirmed agent, which does not take into account the importance of tracking close contacts for the spread of the epidemic. The intervention measures of large-scale vaccination of COVID-19 vaccine and nucleic acid testing have not been taken into account, so the simulation data deviates greatly from the real data.

Therefore, in the current model, the geospatial environment is narrowed down to the urban districts with dense population that are prone to outbreaks, and the municipal districts are divided into six regions according to the actual situation. In order to highlight human self-control and the interaction between autonomous decision-making behavior and the spatial environment, a new government agent is added to the current model, which adjusts the response level in real time based on the current epidemic data of the artificial society, and the resident agent also makes autonomous decisions based on the current response level, deciding whether to flow, as well as the flow speed and target area, and when the prevention and control level is higher, the flow speed of the crowd will be slowed down accordingly. At the same time, the hospital agents, stations, shopping areas, and university agents are added to the current model, and they are mapped to the space environment of the model according to the real geographical location and will adjust the corresponding personalized intervention measures according to the conditions of the municipal district where they are located and the current government agents adjusting the response level and prevention and control policies of major public health emergencies. The crowds around the geographical locations such as hospitals, stations, shopping areas, and universities are denser, and the flow will and speed are also different. In the current model, the visiting rate and the visiting time of the hospital agent will be adjusted according to the current level of prevention and control, and the distance between the agent and the hospital will also affect the visiting time. The resident agents contacted by the latent agents during the flow process will be transformed into close-contact agents, and the infection rate will be affected according to the self-protective measures taken before contact. After being tracked, the close-contact agent and the latent agent will be isolated and regularly tested for nucleic acid, so as to improve the accuracy of the model.

Based on the above improvements, as shown in Table 3, four evaluation indicators are used to evaluate the errors of the two models, namely, the mean absolute error (MAE), the root mean squared error (RMSE), the mean absolute percentage error (MAPE), and the sum of square error (SSE) evaluates the predictive performance of different models in this study. These four indicators are all negative indicators, that is, the smaller the index value is and the smaller the deviation from the true value of the forecast value of the model, the stronger the forecasting ability of the model [32]. The statistical results of each evaluation index of different models are shown in Table 4. The MAE of the current model is reduced by 1.03; the RMSE is reduced by 1.45; the MAPE is reduced by 3.45%, and the SSE is reduced by 173. The simulated index numbers for the current model fitted well with the actual data, and they were significantly improved relative to the existing model, and the accuracy of the model is significantly improved, which was closer to the actual data, thereby indicating that the assessment model for the rapid suppression of SARS-CoV-2 transmission under government control is effective.

## 4. Simulation Scenarios and Analysis of Experimental Results

### 4.1. Government Response Level Adjustment

In the model, the government agent can make real-time adjustments to the response level and prevention and control policies for major public health emergencies based on the number of resident agents in different states. In order to simulate the spread of the virus and the effects of prevention and control under different response levels by the government, the parameters were set for the government agents in this scenario to simulate the spread of the virus under different response levels and with flows into the same number of latent agents from station agents or airport agents at four levels. According to the four emergency response levels defined by the National Health and Wellness Commission of the People’s Republic of China, the government agents responded at the different epidemic levels based on the relevant research findings and the specific parameters set in the emergency plan, as shown in Table 5 [33].

In particular, when the emergency response level of the government agent was at level IV, the prevention and control ability was the weakest. Intervention measures included a self-protection rate of only 20% and a medical treatment rate of 50%. There was no restriction on the flow of people, and the flow rate was the fastest. When the emergency response level was at level III, involving isolation, medical observation, patient treatment, and fixed-point medical treatment for close contacts, the flow of people slowed down, and the SARS-Cov-2 vaccine was researched. At level II, large-scale tracking and isolation, small-scale shutdown and suspension of classes, and the organization of a certain scale of vaccination were added. At level I, large-scale vaccination was conducted, the city was closed, and the population within the effective range of the city stopped moving.

**Figure 8:**
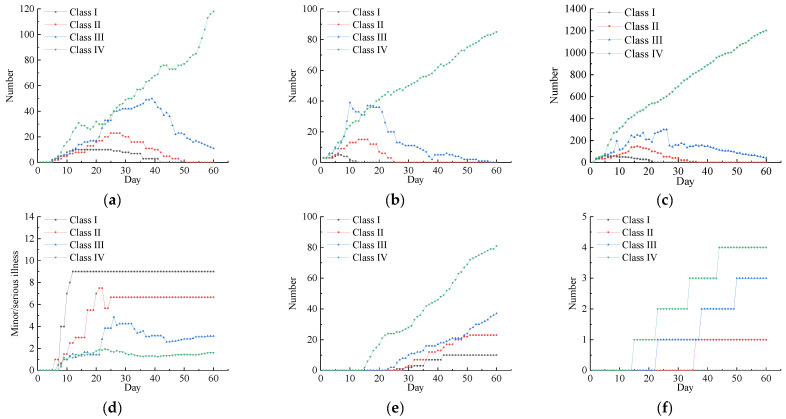
Government response level adjustment scenarios: (**a**) changes in the number of confirmed cases, (**b**) changes in the number of lurkers, (**c**) changes in the number of close contacts, (**d**) mild-to-severe-disease ratio, (**e**) changes in the cumulative number of cured patients, (**f**) cumulative number of deaths.

Each experimental simulation was run 500 times, and the average values of repeated simulations were taken to calculate the experimental results. The experimental results are shown in Figure 8.

According to the experimental data, when the emergency response was at level IV, the number of resident agents in different states continued to rise. When the medical resources were insufficient, the number of agents with diagnosed patient status increased rapidly, and it was difficult to completely cure them. Due to the lack of effective isolation and vaccination, the proportion of severely ill patients was much higher than that of mild-sickness patients. When the emergency response was at level III, the hospital agent adjusted the speed of treatment, strengthened the self-protection awareness of resident agents, slowed down the flow rate, and tracked a certain number of close contacts for effective isolation. The number of confirmed persons, latent persons, and close contacts decreased significantly compared with level IV, but it was difficult to rapidly curb the spread of the epidemic, and the proportion of severely ill patients was not effectively improved. When the emergency response reached level II, the transmission of the virus could be controlled, and the speed of medical treatment and self-protection awareness continued to improve. In addition, half of the population was vaccinated, which decreased the transmission rate rapidly, while the proportion of severely ill cases decreased, the speed of achieving complete cure increased significantly, and the death toll was only one person. When the emergency response reached level I, the closed places were shut down, and classes were suspended to rapidly control the spread of the virus. Large-scale vaccination formed an immune barrier in the population, so the number of latent persons and close contacts decreased significantly, and the confirmed cases were cured in 42 days. The experimental results showed that strong emergency response measures could quickly control the spread of the epidemic, but a strong response level inevitably incurred higher implementation costs and social costs. Therefore, timely evaluations and selecting appropriate prevention and control measures are essential for urban public health management.

### 4.2. Improvements in Disease Control Methods

The effects of intervention control measures under different government response levels were analyzed in this study. It is difficult to measure the effects of prevention and control measures on SARS-CoV-2 in a real artificial society when a small number of intervention measures are superimposed at the same time. Thus, in the next experiment, three different intervention strategies are designed, as shown in Table 6.

Intervention strategy A involved implementing different intervention measures at different times, and intervention strategies B and C also included the first five intervention measures. In intervention strategy C, intervention measure 6 was added, comprising prior vaccination to better control the spread of SARS-CoV-2.

In the model, the willingness of the residents to flow was initially set at 0 (median willingness to flow), and the other parameters were consistent with those described in the implementation part of the simulation model. The corresponding prevention and control measures were implemented according to the different intervention strategies in different simulation time steps. The experimental results are shown in Figure 8.

**Figure 9:**
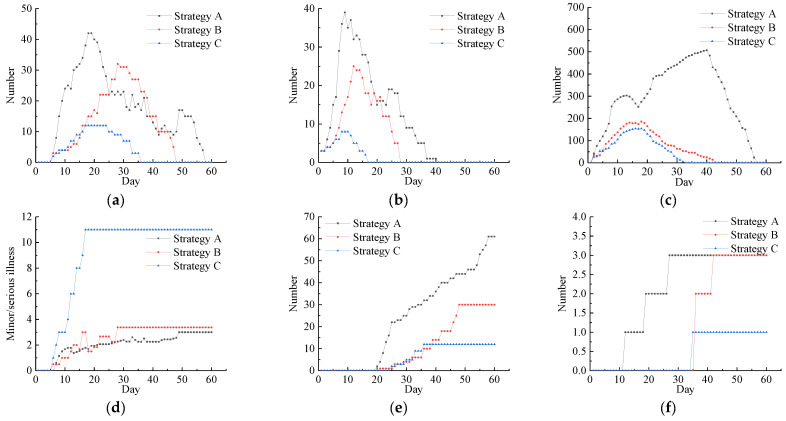
Comprehensive intervention level adjustment scenarios: (**a**) changes in the number of confirmed cases, (**b**) changes in the number of lurkers, (**c**) changes in the number of close contacts, (**d**) mild-to-severe-disease ratio, (**e**) changes in the cumulative number of cured patients, (**f**) cumulative number of deaths.

The awareness of residents about prevention and control was improved due to the effects of the comprehensive intervention measures. The prevention and control of individuals and organizational units was enhanced at all levels, and thus the number of confirmed persons, quarantined persons, close-contact persons, and latent persons decreased significantly and gradually tended towards zero. The number of cured persons increased significantly, and the time required for achieving a cure was shortened. The proportion of patients diagnosed as severe cases decreased. The deaths occurred in people aged over 65 years who had not been vaccinated, thereby indicating that elderly people with complex health conditions were at a high risk of death. The different intervention strategies also affected the increases in the numbers of newly confirmed persons, latent persons, and close-contact persons. Figure 9a–c shows curves representing the changes in the number of resident agents in different states under the three intervention strategies over time. The experimental results obtained under intervention strategies C and B were lower than the total number under intervention strategy A, and the growth rate was higher under strategy A than the other two strategies, thereby indicating the importance of medical treatment, tracking, isolation, and limiting the timeliness of population movements to rapidly curb the spread of the epidemic. As shown in Figure 9d, the proportion with mild disease was much higher under intervention strategy C than strategies A and B, thereby indicating that vaccination was the most effective measure for reducing the risk of severe disease. The simulation results showed that the number of agents in different states was effectively controlled under intervention strategy C, thereby indicating that taking multiple measures at the same time could effectively control the spread of SARS-CoV-2 and that vaccinating a large number of people could gradually build an immune barrier. The factors that affect the spread of SARS-CoV-2 are complex and diverse. Thus, it is difficult to effectively control the spread of SARS-CoV-2 by using a single intervention measure. Therefore, it is essential to implement multiple measures at the same time.

## 5. Conclusions

In the present study, a multi-agent modeling approach based on the current epidemic situation, virus transmission characteristics, real data, and the spatial environment in a realistic artificial society was used to construct a model of rapid SARS-CoV-2 transmission suppression assessment under government control, which can simulate various scenarios and hypotheses and which is suitable not only for traditional SARS-CoV-2 but also for its variant strains. It is another tool for epidemiological modeling and decision-making. The model can import a geospatial map and the corresponding coordinate positions, then adjust the epidemic parameters to be evaluated and simulated through the calculation of the mathematical model; by adding or deleting different types of agents in the agent model library, it can be used to simulate and predict different types of epidemic in different regions. Different from traditional model analysis methods that focus only on macroscopic patterns of epidemic spread, in the model, humans and environmental entities are abstracted as agents, thereby highlighting the effects of human self-control and autonomous behavior, population heterogeneity, and policy changes by environmental entities on suppressing the spread of the virus. This model combines the randomness and spatial effects of epidemic transmission to define crowd contact patterns and interaction behavior rules that allow the agents to make adaptive changes as the environment changes. In order to improve the prediction accuracy of the model, the environmental model was refined, and the intervention measures, such as vaccination, medical treatment, and close-contact tracing, were introduced in combination with the age condition and the self-protection of different reactive agents. In particular, the governmental agent can respond to the changing epidemic situation in real time, and multiple other environmental entities implement different prevention and control measures in a cooperative manner. The effectiveness of multiple combinations of interventions were analyzed and evaluated based on the dynamic interaction feedback mechanism between humans and the spatial environment to simulate virus transmission in complex urban systems and the changes under different interventions. By fitting real data and comparing it with the data from existing models, the accuracy of the model is significantly improved, and it can accurately predict the development of the epidemic in the city within a certain period of time.

We tested the proposed model in two different scenarios, and the simulation results indicated two main findings. First, an adjustment of the response levels and prevention and control policies for major public health emergencies by the government affected the spread of the virus, and stronger emergency response measures could more rapidly control the spread of the epidemic. Second, experimental comparisons of the improved disease control methods showed that implementing different combinations of intervention strategies in the same place at different times was significantly more effective for the prevention and control of SARS-CoV-2. In particular, vaccination was the most effective and economical method for containing the spread of the virus. Therefore, the agile adjustment of governmental prevention and control strategies according to the spread of the epidemic is crucial for controlling the spread of the epidemic, and a “one-size-fits-all” approach to prevention and control should not be adopted at the same time. The effectiveness of policies should be reasonably adjusted according to the actual situation, and governments should have an active role in promoting disease awareness in prevention and control efforts [34].

The model should be improved by conducting further simulation experiments and considering epidemiological theories, as well as subdividing the population and geographical areas and adopting different prevention and control measures according to the epidemic situations in different geographical areas. It is also necessary to study the spread of the virus due to the cross-domain flow of the population, as well as the negative impacts of different prevention and control measures on social and economic development. More precise and normalized prevention and control measures can be explored to provide more effective decision support for ensuring urban health protection.

## Figures and Tables

**Table 1:** Agent behaviors and descriptions.

Agent Behavior	Description of Behavior	Applicable Subjects
Spatial mobility	Resident agents other than quarantined agents and confirmed cases engage in purposeful spatial mobility behavior.	Resident Agent
Status transitions	Under certain conditions, the agents undergo a qualitative state transition, e.g., healthy agents that contact latent agents switch to being close-contact agents.	Resident Agent
Wearing a mask/vaccination	Agents have a certain degree of autonomous decision-making ability and the real-time awareness of current policies and prevention and control levels to take appropriate preventive and control measures.	Healthy-People Agent
Close contact	Contacted agents are converted into close-contact agents by moving through the relevant trajectory to reach healthy people agents within a certain range.	Close-Contact Agent
Viral infection	Close-contact agents are converted into latent agents under certain probabilistic conditions based on preventive and control measures taken prior to close contact, and they infect healthy agents during their movement to make them undergo a state transition.	Latent Agent
Quarantine	Most of the close-contact agents are tracked according to the current level of control to convert them into quarantined agents, which do not have mobile behavior and regularly undergo nucleic acid testing.	Quarantined Agents
Confirmation	Determine the confirmation times for quarantined agents according to the regular nucleic acid detection process and individual indicators, and convert them into confirmed agents.	Diagnosed-Person Agents
Disease progression	The course of the disease is determined by the preventive and control measures taken prior to the onset of the disease, the promptness of treatment after the onset of the disease, and the physical indicators of the person diagnosed.	Diagnosed-Person Agents
Admission of a confirmed case	The confirmed agent is admitted according to the amount of available beds, medical resources, and medical staff.	Hospital Agents
Checking behavior	Mobile resident agents are allowed to move within a designated area after appropriate checks have been carried out on them according to the current public health and safety response level of the government agents.	Stations, Shopping Areas, University Agents
Closure behavior	Prohibit the movement of resident agents within a designated area based on government closure measures according to the current public health and safety response level of the government agents.	Stations, Shopping Areas, University Agents
Adjustment of response levels and prevention and control policies	Adjust the response level and prevention and control policy according to the current number of confirmed, close-contact, and quarantined agents.	Government Agent

**Table 2:** Criteria for classifying government emergency response levels.

Response Level	Basis of Classification
Level IV	The last confirmed agent was discharged from hospital with a cure no less than 14 days ago.
Level III	The number of confirmed agents is 0, or there have been no new confirmed agents for 14 consecutive days.
Level II	New confirmed agents within 14 days, and the cumulative number of confirmed agents is less than 50
Level I	Cumulative number of confirmed agents exceeds 50.

**Table 3:** Evaluation indicators.

Indicators	Name	Formula
MAE	Average absolute error	$MAE=\frac{1}{n}\overset{n}{\underset{t=1}{\sum }}\left|x_{t}-y_{t}\right|$
RMSE	Root mean square error	$RMSE=\sqrt{\frac{1}{n}\overset{n}{\underset{t=1}{\sum }}{\left(x_{t}-y_{t}\right)}^{2}}$
MAPE	Average absolute error percentage	$MAPE=\frac{1}{n}\overset{n}{\underset{t=1}{\sum }}\left|\frac{x_{t}-y_{t}}{x_{t}}\right|\times 100\%$
SSE	Error sum of squares	$SSE=\overset{n}{\underset{t=1}{\sum }}{\left(x_{t}-y_{t}\right)}^{2}$

**Table 4:** Statistical results of each evaluation index of different models.

Model	MAE	RMSE	MAPE (%)	SSE
Available model	1.96	2.71	6.93	221
Improved model	0.93	1.26	3.48	48

**Table 5:** Parameter settings for interventions under four emergency response levels.

Emergency Response Levels	Class IV	Class III	Class II	Class I
Visiting hours/day	4	3	2	1
Attendance rate/%	0.5	0.5	0.7	0.9
Isolation ratio/%	0	0.5	0.7	0.9
Vaccination rate/%	0	0	0.5	0.8
Self-protection rate/%	0.2	0.5	0.8	0.95
Willingness to move/%	0.99	0	−0.5	−0.99

**Table 6:** Components of the intervention strategies.

Intervention Measures	Strategy A	Strategy B	Strategy C
Self-health protection	Day 1	Day 1	Day 1
Wear a mask	Day 1	Day 1	Day 1
Hospital treatment	Day 3	Day 1	Day 1
Close contacts are tracked and isolated.	Day 5	Day 1	Day 1
Limit the flow of people	Day 7	Day 1	Day 1
Vaccine intervention	——	——	Day 1

## Data Availability

Not applicable.

## References

[1] Lai S., Ruktanonchai N.W., Zhou L., Prosper O., Luo W., Floyd J.R., Wesolowski A., Santillana M., Zhang C., Du X. (2020). Effect of non-pharmaceutical interventions to contain COVID-19 in China. Nature.

[2] Yan B., Tang X., Liu B., Wang J., Zhou Y., Zheng G., Zou Q., Lu Y., Tu W. (2020). An Improved Method for the Fitting and Prediction of the Number of COVID-19 Confirmed Cases Based on LSTM. CMC-Comput. Mater. Continua..

[3] Duan W., Fan Z., Zhang P., Guo G., Qiu X. (2015). Mathematical and computational approaches to epidemic modeling: A comprehensive review. Front. Comput. Sci..

[4] Li M.Y., Muldowney J.S. (1995). Global stability for the SEIR model in epidemiology. Math. Biosci..

[5] Ajelli M., Goncalves B., Balcan D., Colizza V., Hu H., Ramasco J.J., Merler S., Vespignani A. (2010). Comparing large-scale computational approaches to epidemic modeling: Agent-based versus structure metapopulation models. BMC Infect. Dis..

[6] Brown S.T., Tai J.H.Y., Bailey R.R., Cooley P.C., Wheaton W.D., Potter M.A., Voorhees R.E., LeJeune M., Grefenstette J.J., Burke D.S. (2011). Would school closure for the 2009 H1N1 influenza epidemic have been worth the cost: A computational simulation of Pennsylvania. BMC Public Health.

[7] Huang J., Zhang L., Liu X., Wei Y., Liu C., Lian X., Huang Z., Chou J., Liu X., Li X. (2020). Global prediction system for COVID-19 pandemic. Sci. Bull..

[8] Leontitsis A., Senok A., Alsheikh-Ali A., Ai N.Y., Loney T., Alshamsi A. (2021). SEAHIR: A Specialized Compartmental Model for COVID-19. Int. J. Environ. Res. Public Health.

[9] Teslya A., Pham T.M., Godijk N.G., Kretzschmar M.E., Bootsma M.C., Rozhnova G. (2020). Impact of self-imposed prevention measures and short-term government-imposed social distancing on mitigating and delaying a COVID-19 epidemic: A modelling study. PLoS Med..

[10] Ayele W., Tesfaye L., Abagero A., Taye G., Abate B., Habtamu T., Kassahun S., Biruk E., Yitayal M., Mulu S. (2021). COVID 19 Epidemic Trajectory Modeling Results for Ethiopia. Ethiop. J. Health Dev..

[11] Yang Z., Zeng Z., Wang K., Wong S.S., Liang W., Zanin M., Liu P., Cao X., Gao Z., Mai Z. (2020). Modified SEIR and AI prediction of the epidemics trend of COVID-19 in China under public health interventions. J. Thorac. Dis..

[12] Epstein J.M., Parker J., Cummings D., Hammond A. (2008). Coupled contagion dynamics of fear and disease: Mathematical and computational explorations. PLoS ONE.

[13] Rahmandad D., Sterman J. (2008). Heterogeneous and network structure in the dynamics of diffusion: Comparing agent-based and differential equation models. Manag. Sci..

[14] Oliver N., Lepri B., Sterly H., Lambiotte R., Deletaille S., De Nadai M., Letouzé E., Salah A.A., Benjamins R., Cattuto C. (2020). Mobile phone data for informing public health actions across the COVID-19 pandemic life cycle. Sci. Adv..

[15] Pan L., Zhang L., Qin S., Yan H., Peng R., Li F. (2021). Study on an Artificial Society of Urban Safety Livability Change. Int. J. Geo-Inf..

[16] Pan L.H., Yang F.Y., Lu F.P., Qin S.P., Yan H.M., Peng R. (2020). Multi-Agent Simulation of Safe Livability and Sustainable Development in Cities. Sustainability.

[17] Pan L.H., Li X.W., Qin S.P., Zhang Q.J., Yan H.M. (2020). A multi-agent model of changes in urban safety livability. SIMULATION: Trans. Soc. Model. Simul. Int..

[18] Lopez A.F., Cárdenas P.F., Ruiz-Canales A., Jimenez F., Portacio A. (2020). A survey on intelligent agents and multi-agents for irrigation scheduling. Comput. Electron. Agric..

[19] Li P. (2011). The Chinese Urban Grid Management Research and Development. Urban Dev. Stud..

[20] Zhan J., Liu Q., Zhang Y.Z., Hao Y., Liang Y., Qu G.B., Zhou Q.F., Jiang G.B. (2020). Preliminary understanding of the novel coronavirus 2019-nCov. Environ. Chem..

[21] Gu R.P., Jia X.Y., Zhao X.L., Wei Z.Q. (2019). Research on Loading, Optimization and Accurate Modeling and Simulation of Civil Aviation Cargo Aircraft. Comput. Simul..

[22] Li Z.Q., Yang H., Zhang K.P., Fu Y.T. (2014). Distributed Model Predictive Control Based on Multi-agent Model for Electric Multiple Units. Acta Atuomatica Sin..

[23] Zhai P., Ding Y.B., Wu X., Long Y.J., Zhong Y.J., Li Y.M. (2020). The Epidemiology, Diagnosis and Treatment of COVID-19. Int. J. Antimicrob. Agents.

[24] Pan L.H., Qin S.P., Li X.W., Lu F.P., Yang Y.F. (2020). COVID-19 multi-agent simulation model for virus prevention and control. J. Syst. Simul..

[25] Feng Y.G., Yu J., Sang J.T., Yang P.B. (2021). Survey on Knowledge-based Zero-shot Visual Recognitio. J. Softw..

[26] Dai L.S., Reng Y.G., Cui Z., Reng Z.P., Zhang X.P., Liu J.C., Chang Y., Xu L.J., Zhou L.L., Tang L. (2020). Taiyuan Bureau of Statistics. Taiyuan Statistical Yearbook.

[27] Zhang M., Xiao J., Deng A., Zhang Y., Zhuang Y., Hu T., Li J., Tu H., Li B., Zhou Y. (2021). Transmission Dynamics of an Outbreak of the COVID-19 Delta Variant, B.1.617.2–Guangdong Province, China, May–June 2021. China CDC Wkly..

[28] Zhang J.X., Xue H.M., Gong Y.X., Qin Q., Ning C.H., Cao L., Cao Y.X. (2021). Analysis of death time of patients with coronavirus disease 2019. J. Xi’an Jiaotong Univ. (Med. Sci.).

[29] World Health Organization (2020). Report of the WHO-China Joint Mission on Coronavirus Disease 2019 (COVID-19). https://www.who.int/publications-detail-redirect/report-of-the-who-china-joint-mission-on-coronavirus-disease-2019-(covid-19).

[30] Chu D.K., Akl E.A., Duda S., Yaacoub S., Schünemann H.J. (2020). Physical distancing, face masks, and eye protection to prevent person-to-person transmission of SARS-CoV-2 and COVID-19: A systematic review and meta-analysis. Lancet.

[31] Haas E.J., Angulo F.J., McLaughlin J.M., Anis E., Singer S.R., Khan F., Brooks N., Smaja M., Mircus G., Pan K. (2021). Impact and effectiveness of mRNA BNT162b2 vaccine against SARS-CoV-2 infections and COVID-19 cases, hospitalizations, and deaths following a nationwide vaccination campaign in Israel: An observational study using national surveillance data. Lancet.

[32] Huang X.J. (2021). Model Construction and Research of Wind Energy Resource Assessment and Wind Speed Prediction Based on Machine Learning.

[33] Central People’s Government of the People’s Republic of China (2006). National Public Health Emergency Response Plan. http://www.gov.cn/gzdt/2006-02/28/content_213129.htm.

[34] Bouchnita A., Jebrane A. (2020). A hybrid multi-scale model of COVID-19 transmission dynamics to assess the potential of non-pharmaceutical interventions. Chaos Solitons Fractals.

